# Evaluation of Titanium Alloys Fabricated Using Rapid Prototyping Technologies—Electron Beam Melting and Laser Beam Melting

**DOI:** 10.3390/ma4101776

**Published:** 2011-10-10

**Authors:** Mari Koike, Preston Greer, Kelly Owen, Guo Lilly, Lawrence E. Murr, Sara M. Gaytan, Edwin Martinez, Toru Okabe

**Affiliations:** 1 Department of Biomaterials Science, Baylor College of Dentistry, Texas A&M Health Science Center, 3302 Gaston Ave., Dallas, TX 75246, USA; E-Mails: mkoike@bcd.tamhsc.edu (M.K.); pgreer@bcd.tamhsc.edu (P.G.); kowen@bcd.tamhsc.edu (K.O.); lguo@bcd.tamhsc.edu (G.L.); tokabe@bcd.tamhsc.edu (T.O.); 2 Metallurgical and Materials Engineering Department, University of Texas at El Paso, El Paso, TX 79968, USA; E-Mails: smgaytan@miners.utep.edu (S.M.G.); emartinez21@miners.utep.edu (E.M.)

**Keywords:** rapid prototyping, titanium alloy, mechanical properties, grindability, corrosion behavior, dental applications

## Abstract

This study characterized properties of Ti-6Al-4V ELI (extra low interstitial, ASTM grade 23) specimens fabricated by a laser beam melting (LBM) and an electron beam melting (EBM) system for dental applications. Titanium alloy specimens were made into required size and shape for each standard test using fabrication methods. The LBM specimens were made by an LBM machine utilizing 20 µm of Ti-6Al-4V ELI powder. Ti-6Al-4V ELI specimens were also fabricated by an EBM using 40 µm of Ti-6Al-4V ELI powder (average diameter, 40 µm: Arcam AB^®^) in a vacuum. As a control, cast Ti-6Al-4V ELI specimens (Cast) were made using a centrifugal casting machine in an MgO-based mold. Also, a wrought form of Ti-6Al-4V ELI (Wrought) was used as a control. The mechanical properties, corrosion properties and grindability (wear properties) were evaluated and data was analyzed using ANOVA and a non-parametric method (α = 0.05). The strength of the LBM and wrought specimens were similar, whereas the EBM specimens were slightly lower than those two specimens. The hardness of both the LBM and EBM specimens was similar and slightly higher than that of the cast and wrought alloys. For the higher grindability speed at 1,250 m/min, the volume loss of Ti64 LBM and EBM showed no significant differences among all the fabrication methods. LBM and EBM exhibited favorable results in fabricating dental appliances with excellent properties as found for specimens made by other fabricating methods.

## 1. Introduction

Rapid prototyping and manufacturing (RP or RPM) is an emerging technology that has revolutionalized product development and fabrication [1]. The method, which is often called layered manufacturing, solid free-form fabrication, and 3D printing, is a significant technological breakthrough in the product manufacturing industry [2]. Compared to the conventional material forming processes, the part is fabricated by additive processes through a gradual-building of solid material from powder layers to the required shape from profiles created using CAD, X-ray computer tomography (CT), magnetic resource imaging (MRI) scanning, *etc*. Since this method requires no fixtures and tooling, a considerable reduction in the cost and lead time can be achieved. The first RP technology emerged as early as 1987 and used a laser for materials processing. More recently developed systems employ an electron beam in sintering and melting materials. In this article, these processing methods are referred to as laser beam melting (LBM) or selective laser melting (SLM) processes; or electron beam melting (EBM) processes. The LBM process is also commonly called selective laser melting (SLM). At first, this method was developed to produce industrial parts has now been considered as an economical processes for the fabrication medical and dental prostheses. A number of articles have been published with respect to the capabilities of these methods, the mechanism of the additive fabrication processes, properties of products made with these technologies, and applications. Typical articles include those by Murr *et al.* [3,4] where titanium and titanium alloys have been considered to make medical and dental prostheses using these additive technologies. Since the use of an inert gas or a vacuum is a necessity for a quality laser and electron beam fabrication, respectively, this is ideal for fabricating titanium components which are easily oxidized in air. The purpose of the present article was to compare the mechanical properties and some other relevant properties for biomedical applications of a titanium alloy (Ti-6Al-4V ELI) fabricated using the LBM and EBM methods. 

## 2. Materials and Methods

### 2.1. EBM and LBM Systems

**Figure 1:**
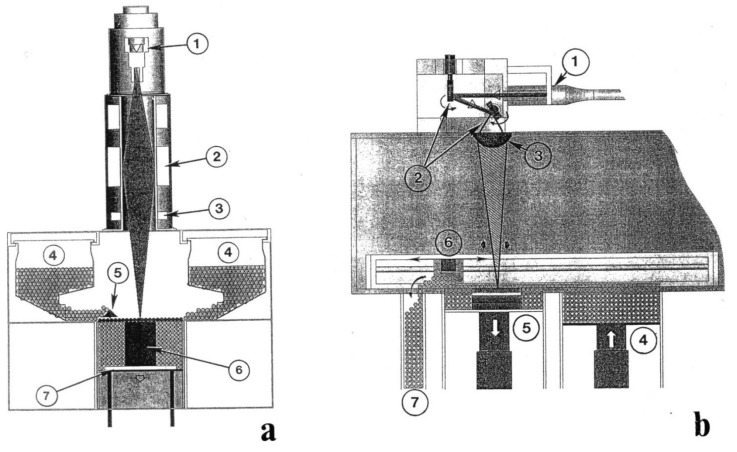
Schematic drawings of an electron beam melting (EBM) system (**a**) and a selective laser melting system (**b**). See text for descriptions. (After Murr *et al.* [4]).

Figure 1 shows comparative schematic views for the EBM (Figure 1(a)) and the LBM (or SLM) (Figure 1(b)) systems. The EBM system in Figure 1(a) utilizes a vacuum environment similar to other electron optical systems such as an SEM. Electrons generated in the gun (1) are focused (2) and electromagnetically scanned. (3) Powder is fed by gravity from cassettes (4) and raked into layers (5) across the build table. The building specimen (6) is lowered on the build table (7) with each layer-melt cycle. The SLM system in Figure 1(b) utilizes a purified gas environment (either argon or nitrogen). The laser beam enters the system at (1), is deflected by a mirror system at (2), and focused in a lens at (3). Powder in a cassette system at (4) is spread by a raking system at (6) over the build platform at (5). Excess powder is reused as shown at (7). 

The specimen compartment houses the powder hoppers to supply the metal powder and the rake or powder distributor to spread the powder during the layering process on a build table, on which a designed object or component are built up layer by layer. 

**Figure 2:**
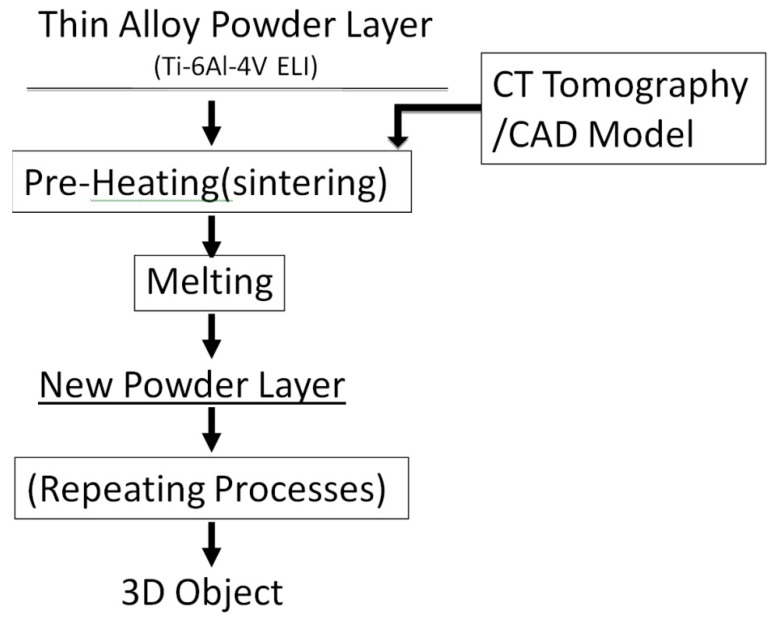
Flow sheet of fabricating 3D object using EBM.

The fabrication of a 3D object goes through several stages as shown in Figure 2 for the EBM process. At first, a CAD model of an object is produced into data formatted in stl. using a digital file obtained by computer tomography (CT scan) of the object with a designed dimension. The equipment builds a metal powder layer which is spread each time at a thickness of approximately 100 µm thick, from the bottom up, by selectively scanning the focused electron beam first, to preheat or sinter specific areas of the metal powder layer as directed by the 3D CAD model. The pre-heating process at lower beam power is needed to lightly sinter the powder to prevent the metal powder from spreading. Then, the same selected areas are melted using full beam power, resulting in the formation of a molten pool and solidifying it into a fully dense layer with a fine-scale microstructure. The table is then lowered, and a new powder layer is spread to continue building the 3D object. In the EBM processing, these three basic processes, powder spreading, pre-heating and melting, are repeated until the 3D object is completely fabricated according to the design. The operation of fabricating a 3D objective in the LBM equipment is basically similar to that for the EBM equipment. However, major differences between two systems, EBM and LBM, are the thickness of the powder layer (100 µm *vs.* 40 µm). The building speed of the LBM system is more rapid than the EBM system (~10^4^ mm/s, respectively). This gives rise to more rapid cooling especially for small size specimens fabricated by LBM. Typical operational conditions for each process are summarized in Table 1.

**Table 1:** Operative conditions for EBM *vs.* LBM systems.

Process	Laser Beam Melting (EOSINT M270)	Electron Beam Melting (Arcam A2)
Environment	argon/nitrogen	
Vacuum/Helium		
Temperature	700 °C	700 °C
Power	5,500 W	7,000 W
Efficiency	High	High
Powder (average size)	20 µm	40 µm
Scan Speed	10^4^ mm/s	10^2^ m/s
Charging	No	Yes
Beam/melt/pool	0.1–0.5 mm	0.2 mm–1.2 mm
Layer thickness	50–100 µm	100 µm
Building speed	7–8 mm/h	6–7 mm/h

### 2.2. Fabrication of Specimens

Each equipment manufacturer specifies an alloy powder that is allowed to build components when the equipment is used. For Arcam A2, the EBM system, the spherical alloy powder of Ti-6Al-4V ELI (ASTM Grade 23, Arcam^®^ AB) with the average particle size of 40 µm was used, whereas for EOSINT M270, the LBM system, the average 20 µm of Ti-6Al-4V ELI spherical particle alloy powder (ASTM Grade 23, EOS) was used. The nominal chemical compositions of each powder are listed in Table 2. Using each alloy powder, for each corresponding equipment, three types of specimens, dumbbell-shaped specimens (20 mm gauge length, 3 mm diameter) for the tensile test; and two kinds of plate specimens (10 mm × 10 mm × 2 mm or 30 mm × 30 mm × 4 mm) for the metallography, the evaluation of corrosion behavior and grindability were fabricated.

**Table 2:** Nominal chemical composition (w/o) of the Ti-6Al-4V ELI powder used for EBM and LBM system.

Process	Al	V	C	Fe	O	N	H	Ti
EBMPowder (ArcamAB)	6.0	4.0	0.03	0.10	0.10	0.01	<0.003	Balance
LBM powder(EOS)	6.0	4.1	0.02	0.10	0.08	0.01	0.0024	Balance

### 2.3. Optical Metallography

Alloy plates (10 mm × 10 mm × 2 mm, n = 2) were metallographically polished and etched using a hydrofluoric acid-based solution [5]. The prepared surfaces were examined using an optical microscope (Epiphot 200, Nikon, Japan). For the LBM and EBM specimens, planes both parallel and perpendicular to the beam direction were examined. 

### 2.4. Electron Microscopy

Plate specimens were sliced into 1 mm of the thickness and mechanically ground and polished up to 1,200 grit. As for the final polish, 0.3 mm diamond paste was used. After polishing and rinsing in the acetone and ethanol, specimens were etched using a solution consisting of 100 mL H_2_O, 2.5 mL HF and 5 mL HNO_3_. The etched samples were observed directly in Hitachi 4800 field-emission scanning electron microscope (FESEM) utilizing secondary electron (SE) image at an accelerating voltage of 20 kV. 

In addition, sections were cut from the LBM and EBM plate specimens and ground and polished to a thickness of approximately 0.2 mm. Standard 3 mm transmission electron microscope (TEM) disc were punched from these thinned sections, dimpled on both sides and electropolished using a Struers Tenupol-5 dual-jet unit using a solution consisting of 0.9 L methanol to which 50 mL H_2_SO_4_ was added. The electropolishing solution was cooled to −10 °C and the electropolishing voltage varied between 15 and 25 V at 5 A to observe the characteristic polishing plateau. These voltage conditions varied for the different microstructures and crystallographic (α + β) mixtures in particular. The resulting electron transparent thin sections were then examined in a Hitachi H-8000 analytical TEM at 200 kV accelerating potential; utilizing a goniometer-tilt stage.

### 2.5. Mechanical Properties

Tensile properties were tested (n = 4) for the dumbbell specimens prepared using the LBM and EBM system and cast specimens at a crosshead speed of 0.25 mm/min at room temperature. Yield strength at 0.2% offset (YS), ultimate tensile strength (TS), modulus of elasticity (E) and percent elongation (El) were determined. Typical testing procedures employed are given in an earlier report [6].

### 2.6. Microhardness

The Vickers microhardness was determined with a 200 g load and 15 s dwell time at more than 300 μm below the surface using a microhardness tester (FM-7, Future Tech, Tokyo, Japan). The microhardness of four randomly chosen areas was determined on two specimens for each metal (n = 8). The procedures for the determination of the hardness are given elsewhere [6]. 

### 2.7. Grindability

Both LBM and EBM plate specimens (30 mm × 30 mm × 4 mm,) were cut into rectangular shapes of 8 mm x 30 mm by slicing them parallel to the direction of the beam, which is perpendicular to the plane of the stacking layers. Both surfaces of the 8 mm × 30 mm plate were ground 0.5 mm so that the resultant thickness of the specimens was reduced to 3 mm. On the other hand, the cast specimens were ground from castings of 3.5 mm × 8.5 mm × 30.5 mm, so that the thickness after the removal of the α-case from the both surfaces was 3 mm. In addition, plates of a similar dimension (3.0 mm × 8.0 mm × 30. mm) were cut from the wrought Ti-6Al-4V ELI pieces and used for test. Grindability (n = 8) was evaluated as volume loss (mm^3^) after grinding for one minute, using a SiC wheel of specimens (13 mm diameter, 1.5 mm thick: 703–120, Brasseler USA, USA) by applying 100 gf at 1,250 m/min. The testing method used for this part of the evaluation was similar to that in a previous study [6]. One of the side walls of 30 mm × 3 mm of the specimens was placed perpendicular to the circumferential surface of the SiC wheel so that the direction of the grinding was parallel to the layer of the LBM and EBM specimen. The volume of metal ground, evaluated by a reduced weight of specimens, was used to compare the grindability among the various metals [7].

### 2.8. Corrosion Behavior

The corrosion characteristics of the specimens were evaluated using a potentiodynamic polarization technique. Evaluation of the corrosion behavior was performed (n = 4) in a modified Tani-Zucchi synthetic saliva maintained at 37 °C as previously reported [8]. Specimen used was plates of 10 mm × 10 mm × 2 mm. A rough surface from the LBM, EBM and wrought plates and the α-case layer of cast specimens were removed. The open circuit potential (OCP: V), polarization resistance (Rp: MΩ•cm^2^), corrosion current density (I_corr_: A/cm^2^) and passivation current density at 500 mV (I_pass_: A/cm^2^) were evaluated using a potentiostat (Model 273; EG&G Princeton Applied Research, Princeton, NJ, USA). For the potentiodynamic corrosion tests, the open circuit potential was evaluated up to 16 hours in the aerated electrolyte. Following the OCP measurement, determination of linear polarization and cathodic polarization were conducted in aerated conditions and anodic polarization in deareated conditions over the ranges and scanning rate as previously reported [8]. A summary of the experimental conditions is given in Table 3.

**Table 3:** Experimental conditions for determination of corrosion behavior.

Methods	Atmosphere	Potential ranges (mV)	Scan rate (mV/sec)	Corrosion parameters
Open-circuit potential (OCP)	-	-	-	OCP: **OCP** (mV) 16 h
Linear polarization	Aerated (Air + 10% CO_2_ )	−8 < OCP to OCP < +8	0.1	Polarization resistance: **R_P_** (MΩ·cm^2^)
Potentiodynamic cathodic polarization	-	OCP to 300 < OCP	0.167	Cathodic Tafel slope: **β_c_** (V/decade)
Potentiodynamic anodic polarization	Deaerated (N_2_ + 10% CO_2_ )	20 0< OCP to 2000 > OCP	0.167	Anodic Tafel slope: β_a_ (V/decade) Corrosion current density: I_corr_ (A/cm^2^) I_corr_ = β_a_β_c_/2.3 R_P_ (β_a_+β_c_) Pasive current density at 500 mV: I_pass_ (A/cm^2^)

### 2.9. Statistical Analysis

The results for all tests, except for corrosion testing, were analyzed using one-way ANOVA and the Tukey’s test at α = 0.05. The data from the corrosion tests were statistically analyzed by Kruskai-Walls H test at a significance level of α = 0.05.

## 3. Results

### 3.1. Exterior Appearance

**Figure 3:**
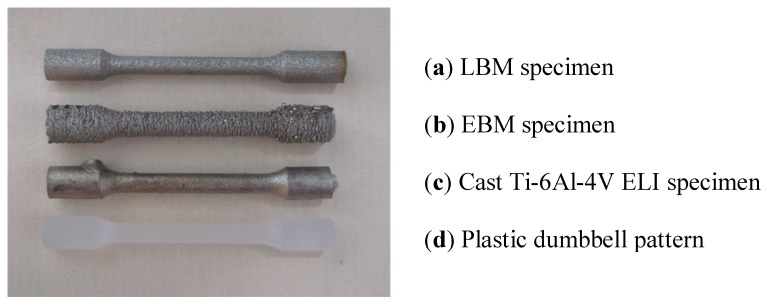
Exterior appearance of (**a**) an LBM specimen; (**b**) an EBM specimen; (**c**) a cast Ti-6Al-4VELI specimen; and (**d**) a plastic dumbbell pattern.

**Figure 4:**
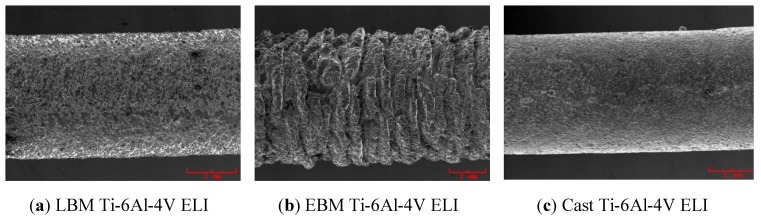
Enlarged views of (**a**) an LBM specimen; (**b**) an EBM specimen; (**c**) a cast Ti-6Al-4V.

Figure 3 compares the external appearances of a LBM-fabricated Ti-6Al-4V ELI specimen, an EBM-fabricated Ti-6Al-4V ELI specimen, a cast specimen and a plastic dumbbell pattern used as the template for preparing a mold for casting the tensile specimens. In Figure 4, an enlarged view of the gauge section of the LBM, EBM and cast dumbbell specimen are also included. The EBM fabricated specimen has a much rough surface with a rippled, exterior appearance.

### 3.2. Microstructures by Optical Microscopy 

**Figure 5:**
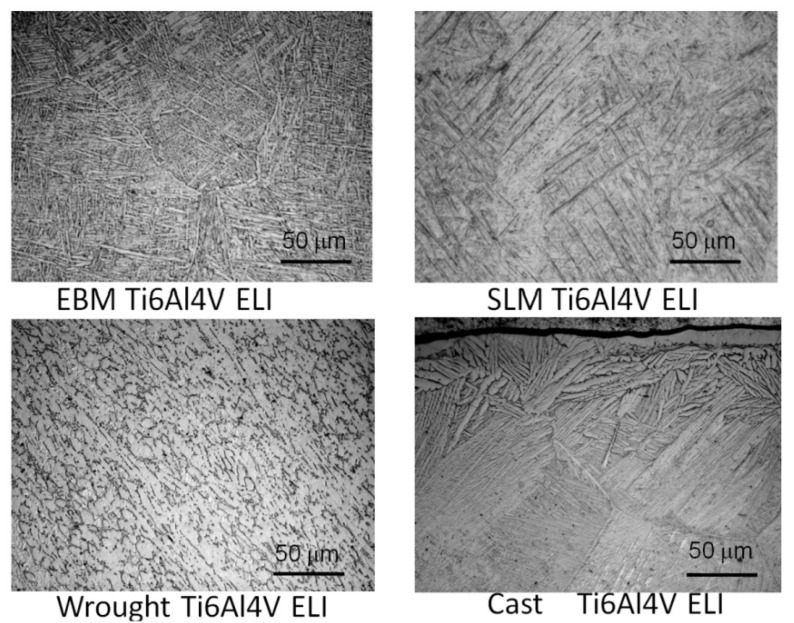
Typical microstructure of (**a**) an LBM specimen; (**b**) an EBM specimen; (**c**) a cast Ti-6Al-4V ELI specimen and (**d**) a wrought specimen.

The microstructures of all those four type of tested alloys shows in Figure 5. Compared to the microstructure of an interior area of the cast specimens, which is away from the α-case layer, all other three types of the specimens, LBM, EBM and wrought specimens all had much finer α-β lamellar structures. The lamellar structure in the cast specimen was much courser than others.

### 3.3. Microstructures by Electron Microscopy

**Figure 6:**
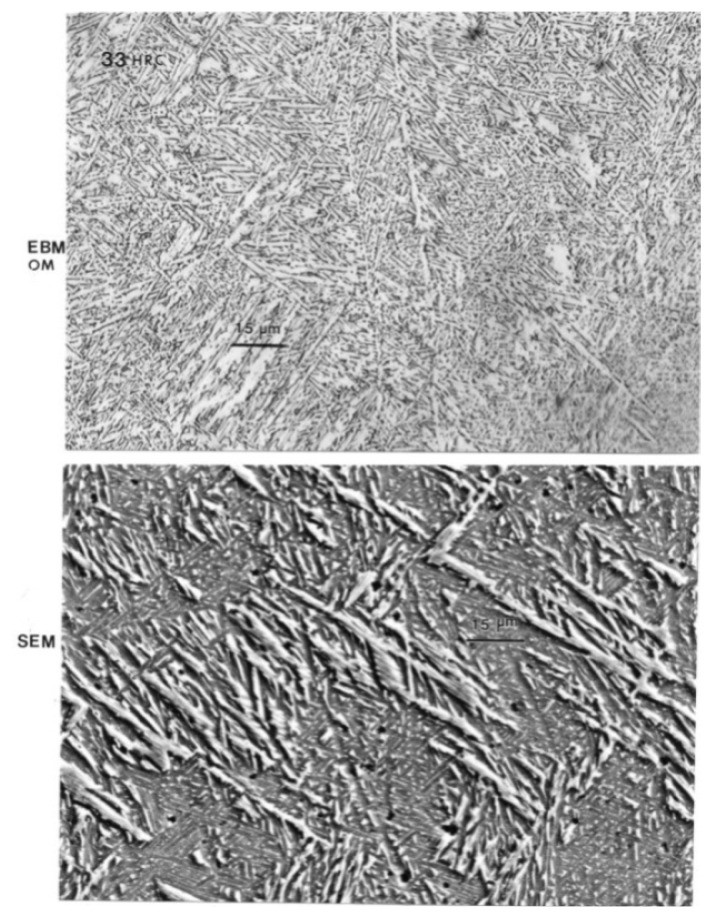
(EBM) Top shows optical (light) micrograph for α-Ti-6Al-4V microstructure. Dark (or black) is β. Bottom shows SEM view of EBM structure as in top. Prominent acicular α-plates.

**Figure 7:**
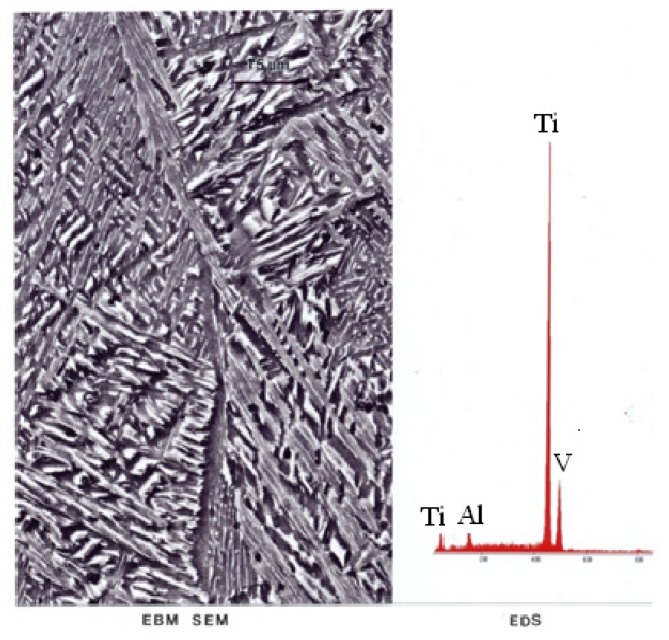
SEM and EDS for EBM Ti-6Al-4V.

**Figure 8:**
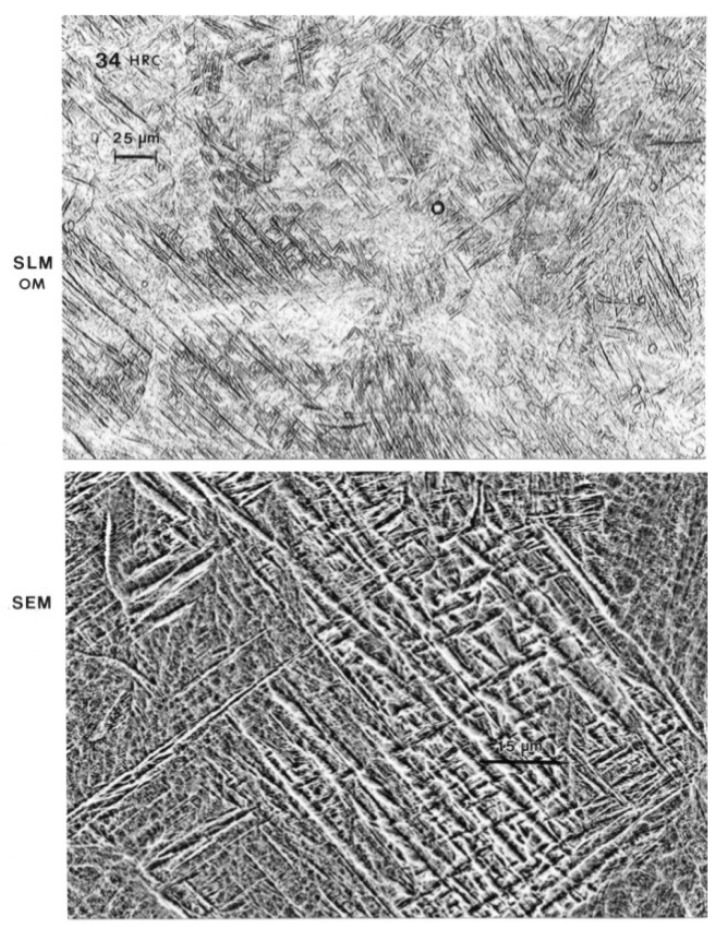
(SLM) Top view shows optical (light) micrograph with primarily α′martensite. Bottom views SEM view.

**Figure 9:**
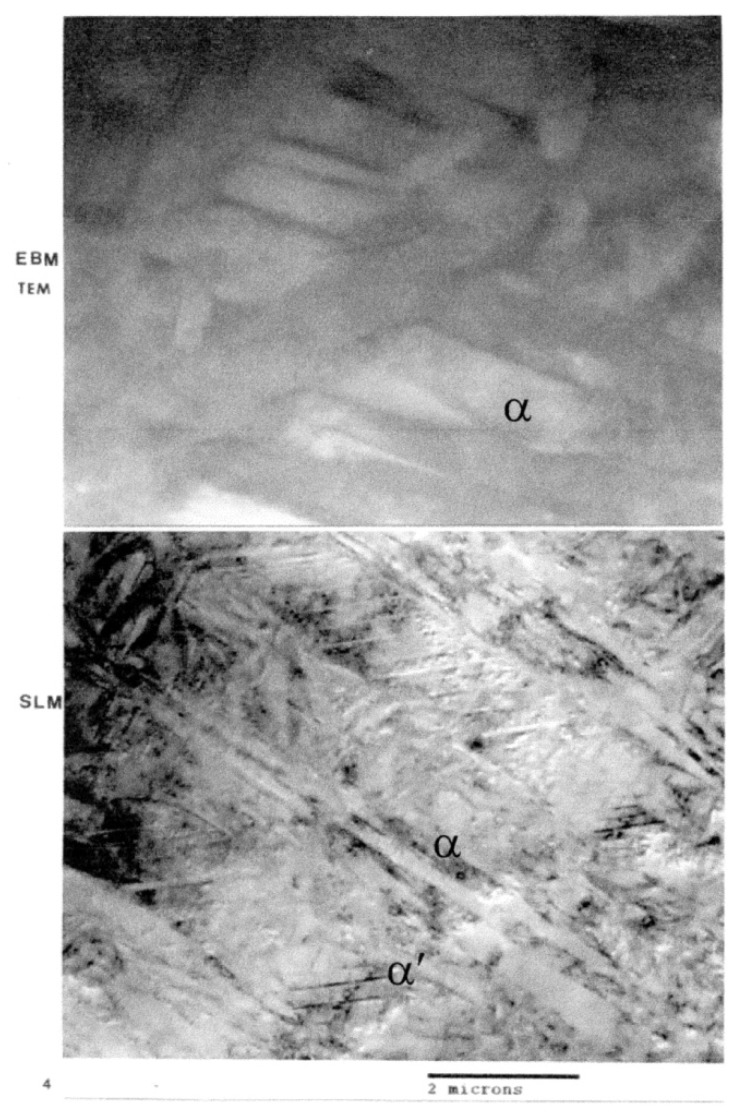
TEM views of EBM-α-phase and SLM mixed α-α′ (with some twinning in α).

**Figure 10:**
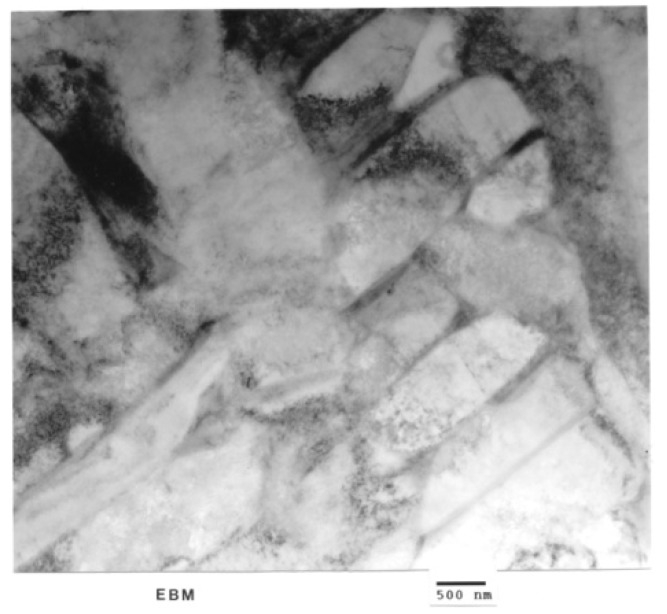
TEM of EBM showing α-phase in Ti-6Al-4V. Dark areas separating α-phase grains is β.

**Figure 11:**
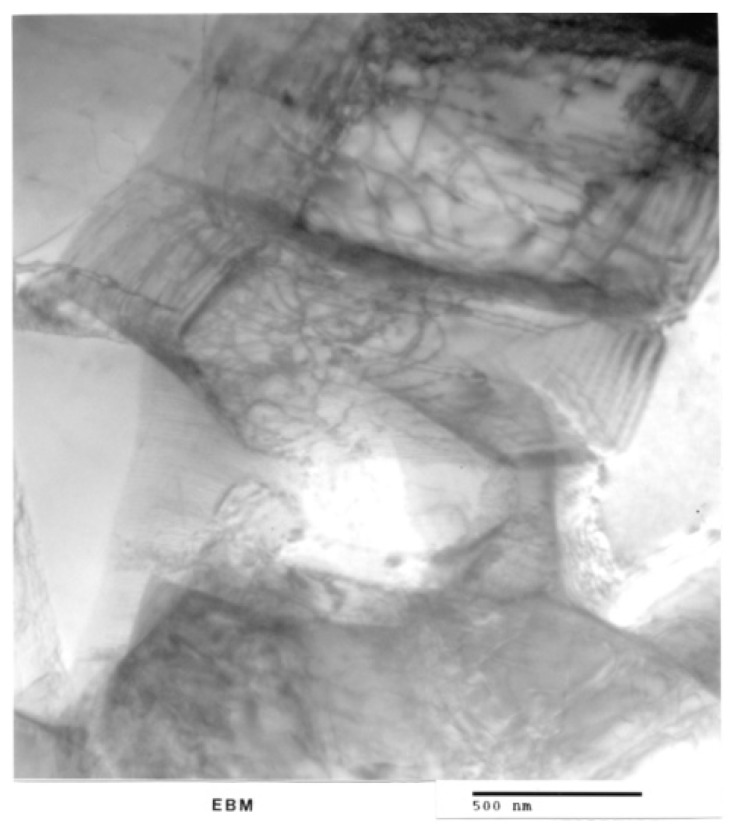
Magnified (TEM) view of α-grains in EBM Ti-6Al-4V sample showing dislocations. α-phase grains are inclined to the specimen surface. Average grain size is ~800 nm (0.8 µm).

**Figure 12:**
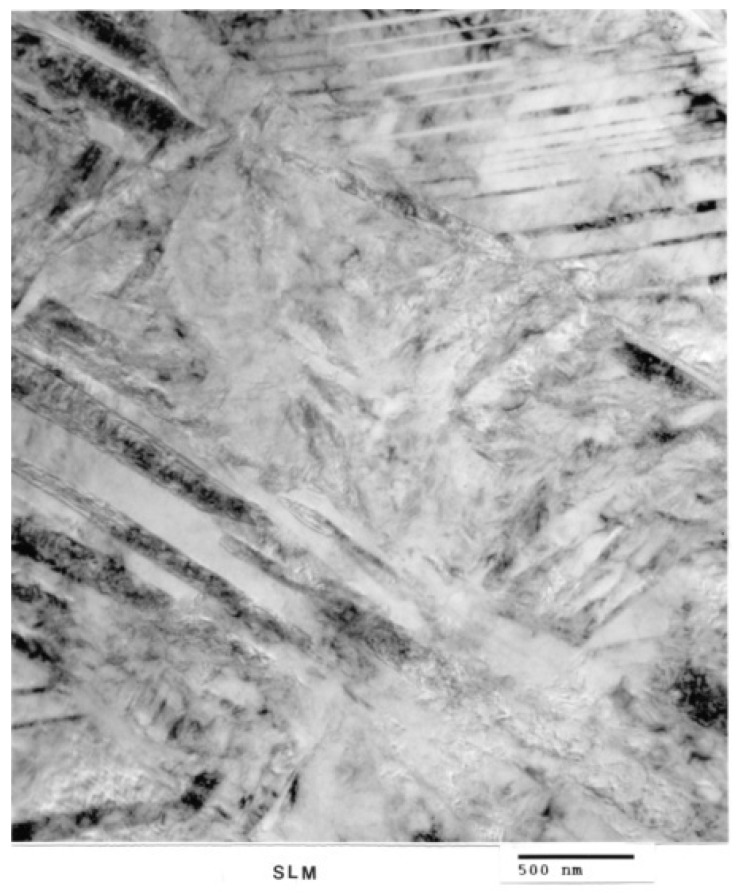
TEM view of SLM α-α′martensite and twinned α.

Figure 6(a) shows optical (light) micrograph for α-Ti-6Al-4V microstructure of EBM specimen. The dark (or black) in this picture is β. Bottom picture of Figure 6(b) shows SEM view of EBM structure as in top, indicating prominent acicular α-plates. The SEM and Spectrum resulting from EDS analysis for EBM Ti-6Al-4V specimen are shown in Figure 7. On the other hand, top view in Figure 3 shows optical micrograph with mixture of α-phase and α′martensite in the LBM specimen. Bottom view in Figure 8 is corresponding SEM view. Figure 9 indicates TEM views of EBM α-phase, while that of LBM consists of mixed α-α′ (with some twinning in α). Figure 10 is the TEM pictures showing that EBM specimen has α-phase in Ti-6Al-4V, and dark areas separating α-phase grains is β. Figure 10 is a magnified TEM view of α-grains in EBM Ti-6Al-4V specimen, showing dislocations. The α-phase grains in Figure 11 are inclined to the specimen surface. Average grain size is ~800 nm (0.8 µm). Figure 12 is a TEM view of α-α′martensite and twinned α on the LBM specimen.

### 3.4. Mechanical Properties

**Figure 13:**
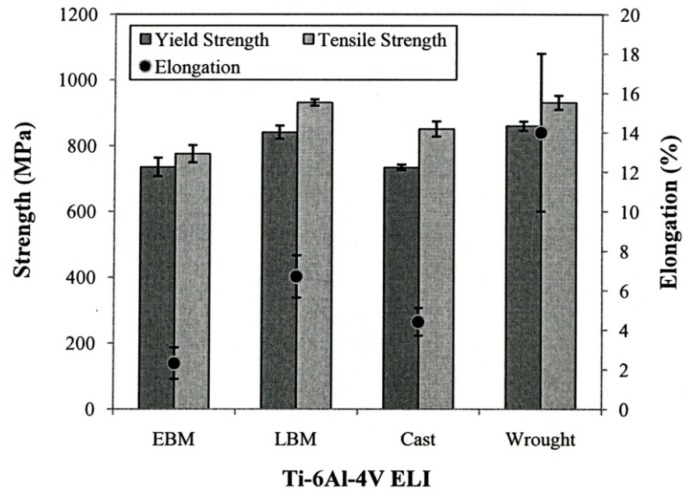
Mechanical properties of metals using different fabrication methods.

**Figure 14:**
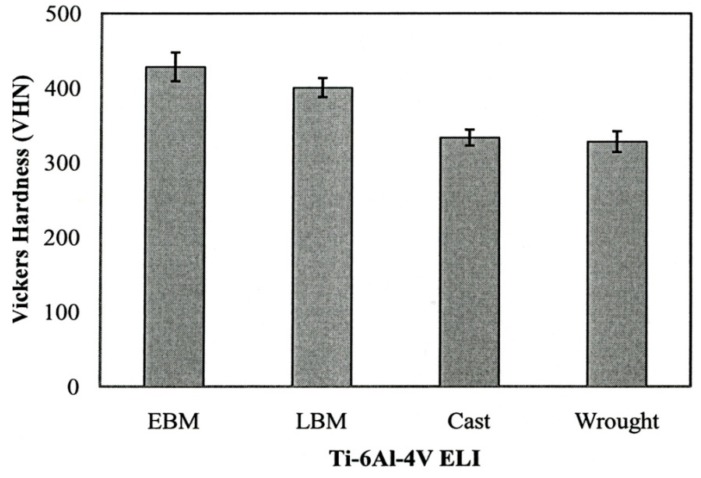
Bulk hardness of Ti-6Al-4V ELI specimens using different fabrication methods.

Figure 13 summarizes the yield strength, tensile strength and elongation of each type of Ti-6Al-4V ELI specimens. The mechanical properties for the wrought specimens were obtained from manufacturer’s data sheet. The strength of the LBM and wrought specimens were similar whereas those of the EBM specimens were somewhat lower than those two specimens. In the properties of the cast specimens, note that the yield strength is the lowest among all types of specimens (p < 0.05). Figure 14 shows the measured interior, bulk microhardness numbers for each type of alloy specimens. The hardness of both the LBM and EBM specimens was similar and roughly 25% higher than that of the cast and wrought alloys (p > 0.05). However, as shown in the optical micrographs in Figure 6(a) and Figure 8(a), the EBM-product hardness measured using a Rockwell C-scale indenter is slightly lower than the SLM-product hardness: 33 HRC *versus* 34 HRC. This is also reflected in the mechanical property data in Figure 13 where both the yield stress and the UTS are roughly 11% higher for the SLM product than the EBM product. This is somewhat reversed from the micorindentation hardness comparisons shown in Figure 14. The elongations for both the EBM and LBM (SLM) products are low in contrast to wrought Ti-6Al-4V in particular, and the EBM product elongation is only about 2%. This is in contrast to elongations of more than 12% reported earlier by Murr *et al.* [9]. Correspondingly, the yield and UTS were more than 30% higher for EBM-fabricated products (Murr *et al.*, 2010 [9]). 

These “variances” are due in part to the fact that the oxygen content for ELI-Ti-6Al-4V powders utilized in this comparative study for EBM *versus* SLM (LBM) varied significantly. The nominal oxygen content for powder is ~0.12% oxygen, but the recycled EBM powder exhibited an oxygen level of 0.34%, roughly 3 times the nominal content. Correspondingly, the LBM powder contained only 0.14% oxygen, and this is in contrast to 0.11% oxygen for the wrought product. Consequently, the interstitial oxygen present in the EBM product is probably responsible for the higher microindentation hardness shown in Figure 14 and the significantly degraded mechanical properties shown in Figure 13.

### 3.5. Grindability

**Figure 15:**
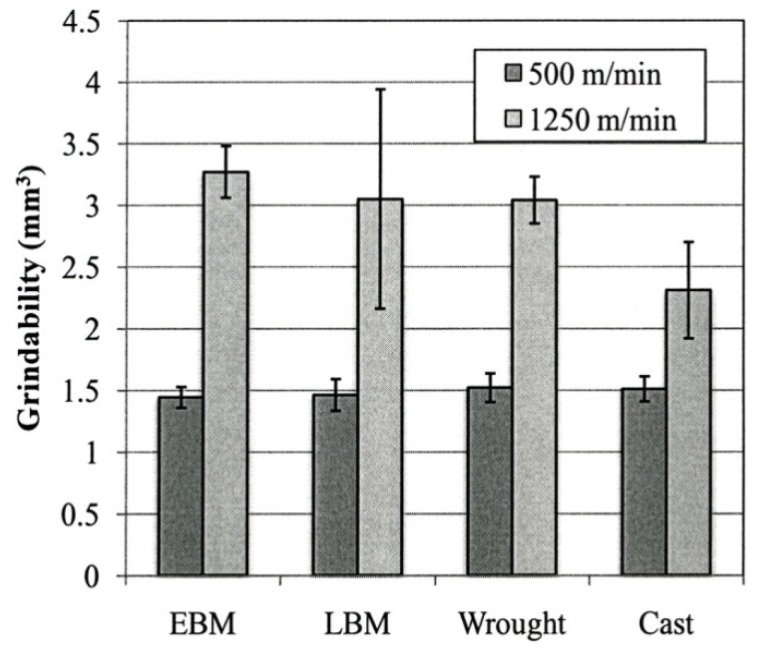
Grindability of Ti-6Al-4V ELI specimens using different fabrication methods.

The grindability for each type of the Ti-6Al-4V ELI specimens for the wheel speeds of 500 and 1.250 m/min are shown in Figure 15. For the higher grindability speed at 1.250 m/min, the grindability of the cast specimen was significantly lower when compared to that of the other specimens (p < 0.05). On the other hand, at 500 m/min, no significant difference was found among all types of the specimens tested (p > 0.05). 

### 3.6. Corrosion Behavior

Table 4 summarizes the corrosion parameter determined specimen using the different fabrication methods. No significant differences were seen in the OCP and the passivation current density among all the fabrication methods. On the other hand, the Kruskai-Walls H test shows significant differences in the polarization resistance (R_p_; p = 0.4) and the corrosion current density (I_corr_: p = 0.2) among all the fabrication methods. The LBM and wrought specimens showed better corrosion resistance compared to that of the EBM specimens and cast specimens. This is certainly related to the fact that in the EBM and cast specimens the α-phase dominates, the acicular α plates are larger than the α′-martensite in the SLM components. In the wrought specimens, there is considerably more β than in the EBM or cast specimens, and the α and β regions are smaller as shown in Figure 5(d).

**Table 4:** Corrosion characteristics of specimens tested.

	OCP (mV)		Rp (MW)		icorr (nA/cm^2^)		ipassive (nA/cm^2^)	
LBM	−239	(18) ^a^	1.81	(0.5) ^a^	23	(8) ^a^	870	(179) ^a^
EBM	−246	(81) ^a^	0.44	(0.3) ^b^	199	(90) ^b^	1645	(583) ^a^
Cast	−243	(18) ^a^	0.75	(0.7) ^b,c^	199	(158) ^b^	1137	(752) ^a^
Wrought	−159	(18) ^a^	1.36	(0.5) ^a,c^	49	(25) ^a^	837	(249) ^a^

Values are means (one standard deviation) for properties of tested alloys. Identical letters indicate no statistical differences (p > 0.05).

## 4. Discussion

In the present study, the microstructures and mechanical properties, grindability and corrosion behavior of the Ti-6Al-4V ELI specimens fabricated by different layer manufacturing techniques were investigated along with conventional cast and wrought fabrication technologies. The Ti-6Al-4V ELI specimens were prepared using newly introduced rapid prototyping machines, a laser beam melting (LBM) or SLM system and an electron beam melting (EBM) system. The tensile data of the LBM and EBM fabricated specimens were compared with those of cast and wrought Ti-6Al-4V ELI. It was interesting to examine the microstructures of all those four types of tested specimens. Compared to the microstructure of an interior area of the cast specimens, which is away from the α-β lamellar structures. The lamellar structure in the cast specimen was much courser than others. 

**Figure 16:**
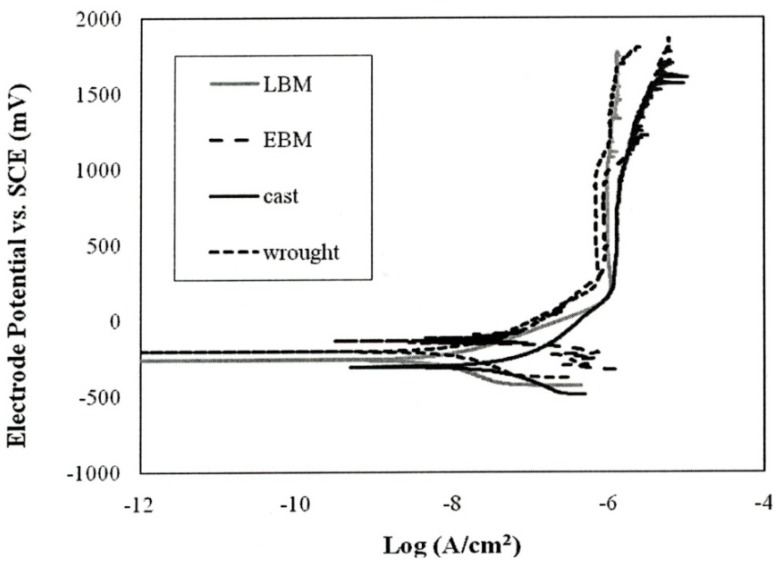
Typical anodic polarization behavior of Ti-6Al-4V ELI specimens using different fabrication methods.

While differences in microstructures for the EBM and SLM-fabricated products as shown in Figure 5, Figure 6, Figure 7, Figure 8, Figure 9, Figure 10, Figure 11, Figure 12 and Figure 13 can account for some variances in properties and performance, differences in oxygen content complicate this issue as noted earlier. It is, however apparent, as noted by Murr *et al.* [9], that LBM fabrication of Ti-6Al-4V products, especially having smaller dimensions, produce more rapid cooling than EBM fabrication, resulting in transformation to α′martensite in various proportions. This microstructure refinement appears to have a significant effect on the corrosion potential which may be especially significant for dental applications. However, if sufficient care is taken to utilize only low oxygen content powder for EBM fabrication, the microstructures may be manipulated to exhibit better mechanical properties and other performance features implicit in Figure 13, Figure 14, Figure 15 and Figure 16. 

It is interesting to note that for the manufacture of small dental implant products, there seems to be little advantage of one melt process over the other. Figure 13 illustrated a small increment of strength for SLM processing over EBM processing while Figure 14 indicated a small hardness increase of EBM fabrication over SLM fabrication. Moreover, both EBM and SLM processing exhibited harder products than wrought or cast products (Figure 14). In contrast, there was no difference in grindability for EBM or SLM products and no corrosion difference. Fundamentally, fine α΄-martensite produced by SLM processing, and this similarity in microstructure behavior is consistent with the mechanical property responses noted above. Laser melting has been shown to allow finer surface structure control than electron beam melting, and this may be a concern for the production of dental implants in particular. 

Recently, Murr *et al.* [9] have shown that open-cellular structures and complex cellular (or porous)-solid monoliths can be fabricated by EBM for potential biomedical implant applications. This could include dental implants which could be fabricated with low density, porous stems with low stiffness (or Young’s modulus) for bone stress shielding reduction. 

## 5. Conclusions

The Ti-6Al-4V ELI specimens fabricated using the electron beam melting (EBM) or the laser beam melting (LBM) systems had a sound microstructure and mechanical properties. Grindability was found to be comparable to those of wrought and cast Ti-6Al-4V ELI specimens. These newly introduced metal-fabrication techniques can be used in preparing numerous types of medical and dental prostheses. Further studies are needed to thoroughly examine specimens prepared at various fabrication conditions to develop optional parameters for properties testing, including dimensional accuracy, corrosion characteristics, fatigue properties, *etc*. 

## References

[1] Das S., Wohlert M., Beaman J.J., Bourell D.L. (1998). Producing metal parts with selective laser sintering/hot isostatic pressing. J. Manag..

[2] Lü L., Fuh J.Y.H., Wong Y.S. (2001). Laser-Induced Materials Sand Processes for Rapid Prototyping.

[3] Murr L.E., Esquivel E.V., Quinones S.A., Gaytan S.M., Lopez M.I., Martinez E.Y., Medina F., Hernandez D.H., Martinez E., Martinez J.L. (2009). Microstructures and mechanical properties of electron beam-rapid manufactured Ti-6Al-4V biomedical prototypes compared to wrought Ti-6Al-4V. Mater. Char..

[4] Murr L.E., Quinones S.A., Gaytan S.M., Lopez M.I., Rodela A., Martinez E.Y., Hernandez D.H., Martinez E., Medina F., Wicker R.B. (2009). Microstructure and mechanical behaviorof Ti-6Al-4V produced by rapid-layer manufacturing, for biomedical applications. J. Mech. Behav. Biomed. Mater..

[5] ASM Handbook Committee (1985). Metals Handbook.

[6] Koike M., Ohkubo C., Sato H., Fujii H., Okabe T. (2005). Evaluation of cast Ti-Fe-O-N alloys for dental applications. Mater. Sci. Eng. C.

[7] Koike M., Chan K.S., Okabe T., Gungor M.H., Imam M.A., Froes F.H. (2007). Dental titanium casting at baylor college of dentistry—Update. Innovations in Titanium Technology.

[8] Koike M., Cai Z., Fujii H., Brezner M., Okabe T. (2003). Corrosion behavior of cast titanium with reduced surface reaction layer made by a face-coating method. Biomaterials.

[9] Murr L.E., Gaytan S.M., Medina F., Martinez E., Martinez J.L., Hernandez D.H., Machado B.I., Ramirez D.A., Wicker R.B. (2010). Characterization of Ti-6Al-4V open cellular foams fabricated by additive manufacturing usingelectron beam melting. Mater. Sci. Eng. A.

